# Supplementary material for: GPX1 and RCN1 as New Endoplasmic Reticulum Stress-Related Biomarkers in Multiple Sclerosis Brain Tissue and Their Involvement in the APP-CD74 Pathway: An Integrated Study Combining Machine Learning and Multi-Omics
Source: Int J Mol Sci. 2025 Jun 29;26(13):6286. doi: 10.3390/ijms26136286 (PMC12249644; doi:10.3390/ijms26136286)
Supplement: Supplementary file 1 [file ijms-26-06286-s001.zip › Supplementary Figure.pdf]

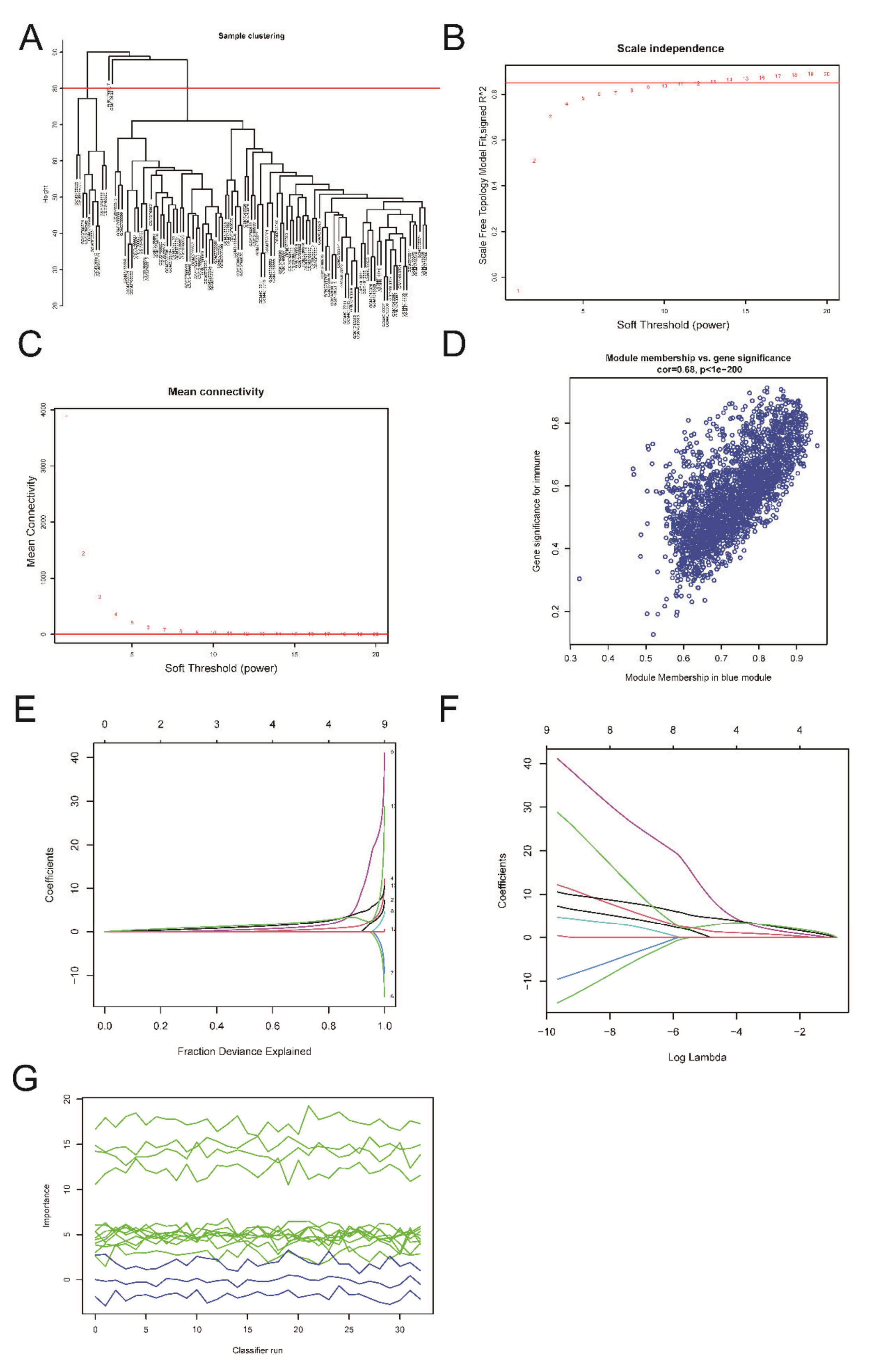

Supplementary Figure S1. (A) Remove outlier samples based on height greater than 80. (B) The panel displays the analysis of the scale-free fitting index with multiple soft-

thresholding powers (β). (C) The panel displays the analysis of average connectivity with multiple soft-thresholding powers. (D) Plot showing the correlation between module membership and gene significance within the module. (E, F) LASSO regression with 10-fold cross-validation, incorporating penalty coefficient adjustment. (G) The working process of Boruta.

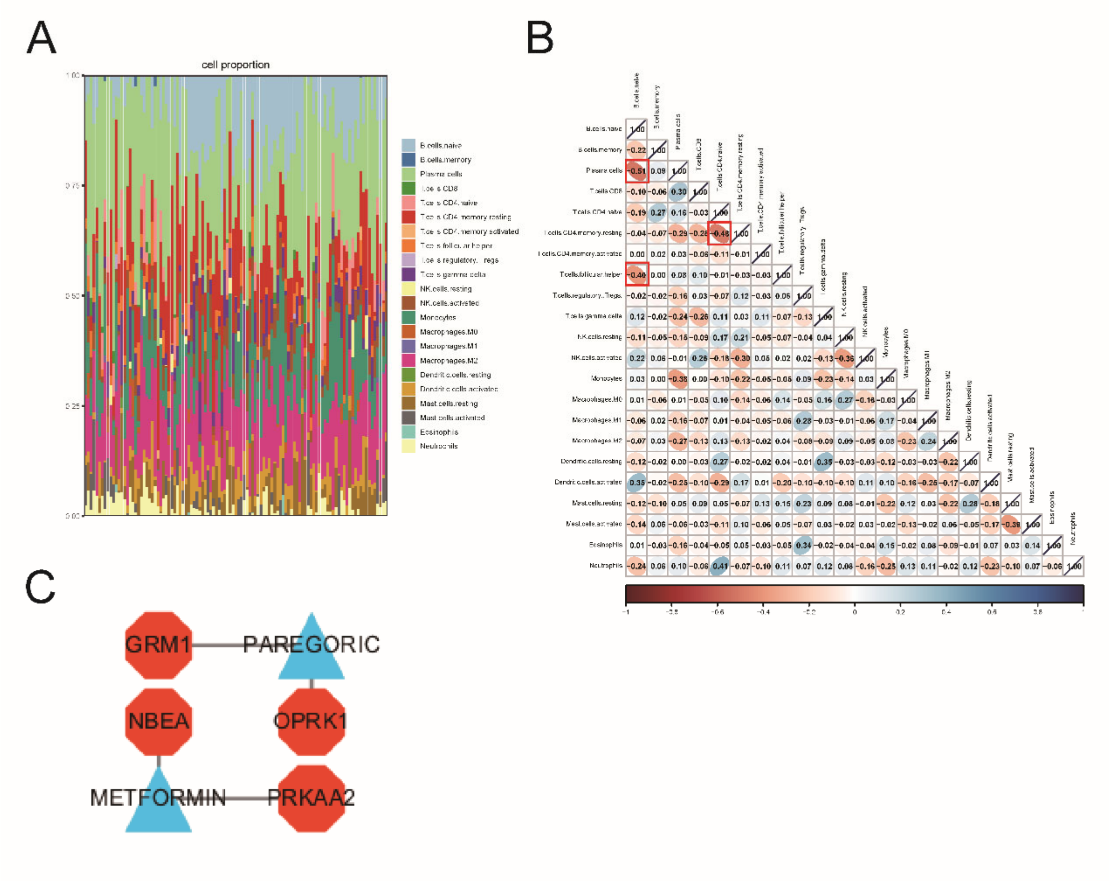

Supplementary Figure S2. (A) The stacked bar chart of immune cell proportions. (B) The correlation heatmap of immune cells. (C) Drugs associated with highly expressed genes in Consensus Clustering Group A.

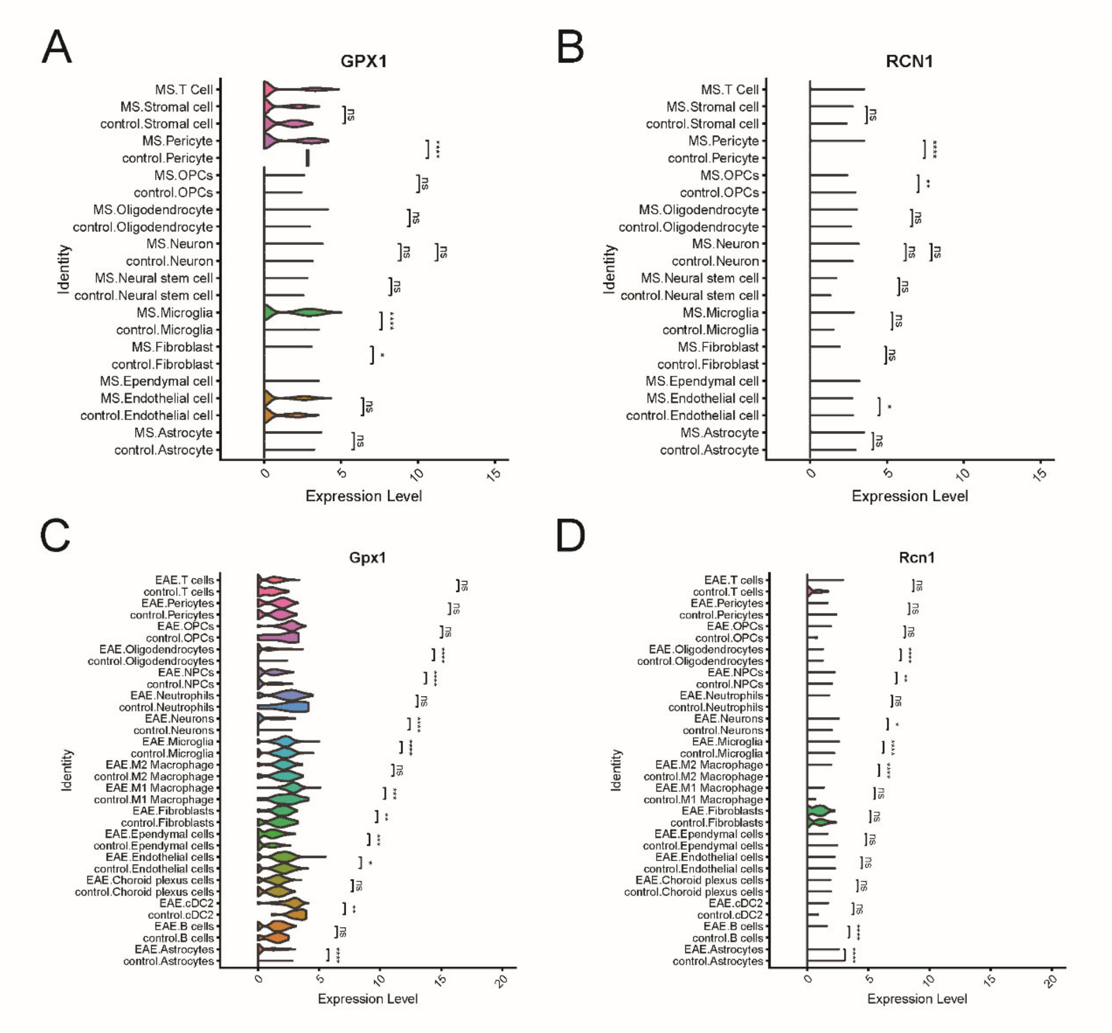

Supplementary Figure S3. (A, B) Expression of *GPX1* and *RCN1* in different cells between the MS and control groups in GSE118257. (C, D) Expression of *GPX1* and *RCN1* in different cells between the EAE and control groups in GSE199460.

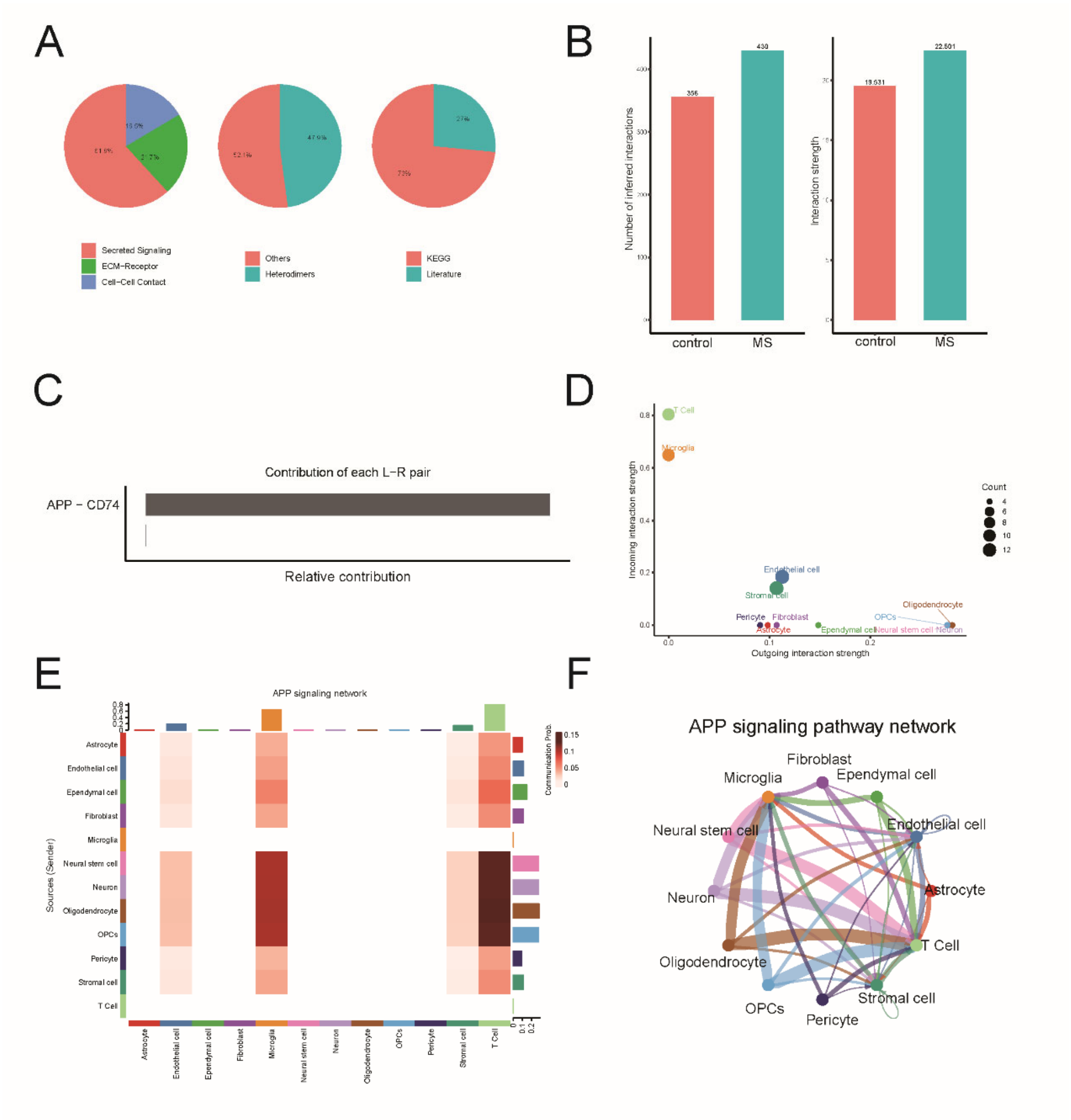

Supplementary Figure S4. (A) The sources of the pathway datasets. (B) Bar chart comparing the strength and quantity of cell communication between MS and the control. (C) Contribution of the APP-CD74 ligand-receptor pair. (D-F) The scatter plot, heatmap, and chord diagram of the APP-CD74 pathway.

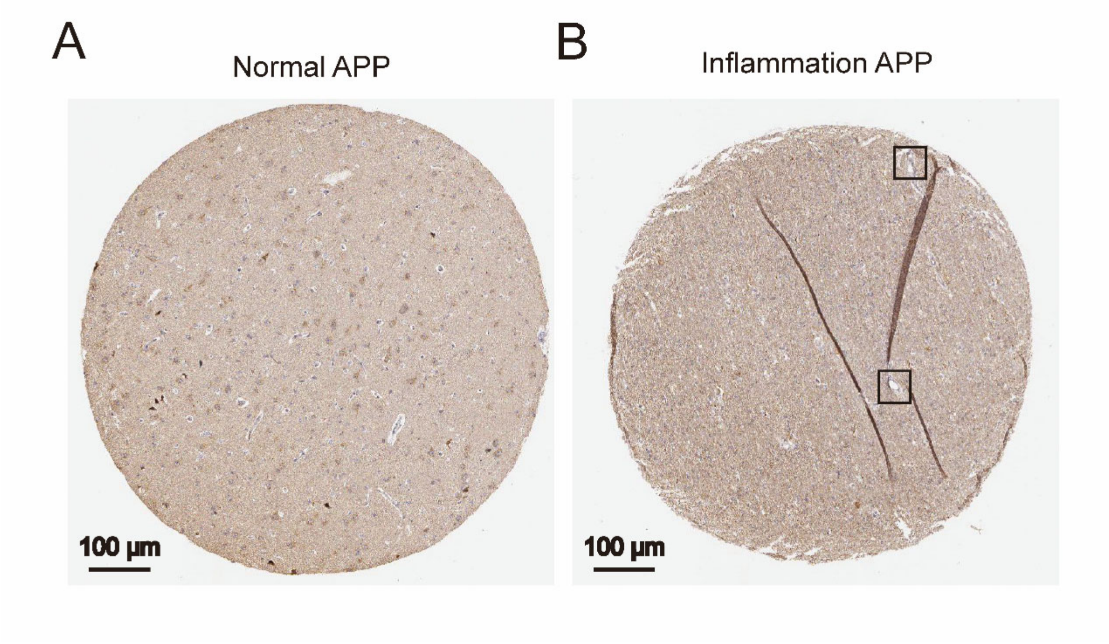

Supplementary Figure S5. (A) The expression of APP protein in normal brain tissue. (B) The expression of APP protein in inflamed brain tissue.

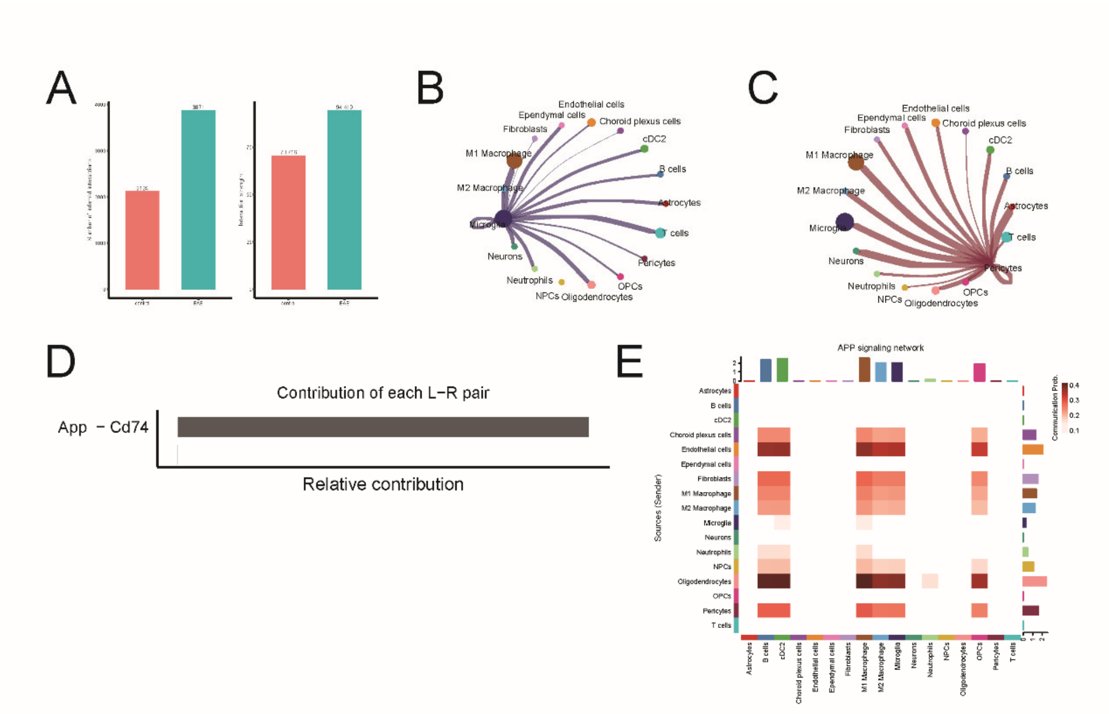

Supplementary Figure S6. (A) Bar chart comparing the strength and quantity of cell communication between EAE and the control. (B, C) The chord diagram and heatmap of the APP-CD74 pathway. (D) Contribution of the APP-CD74 ligand-receptor pair. (E) The heatmap of the APP-CD74 pathway.

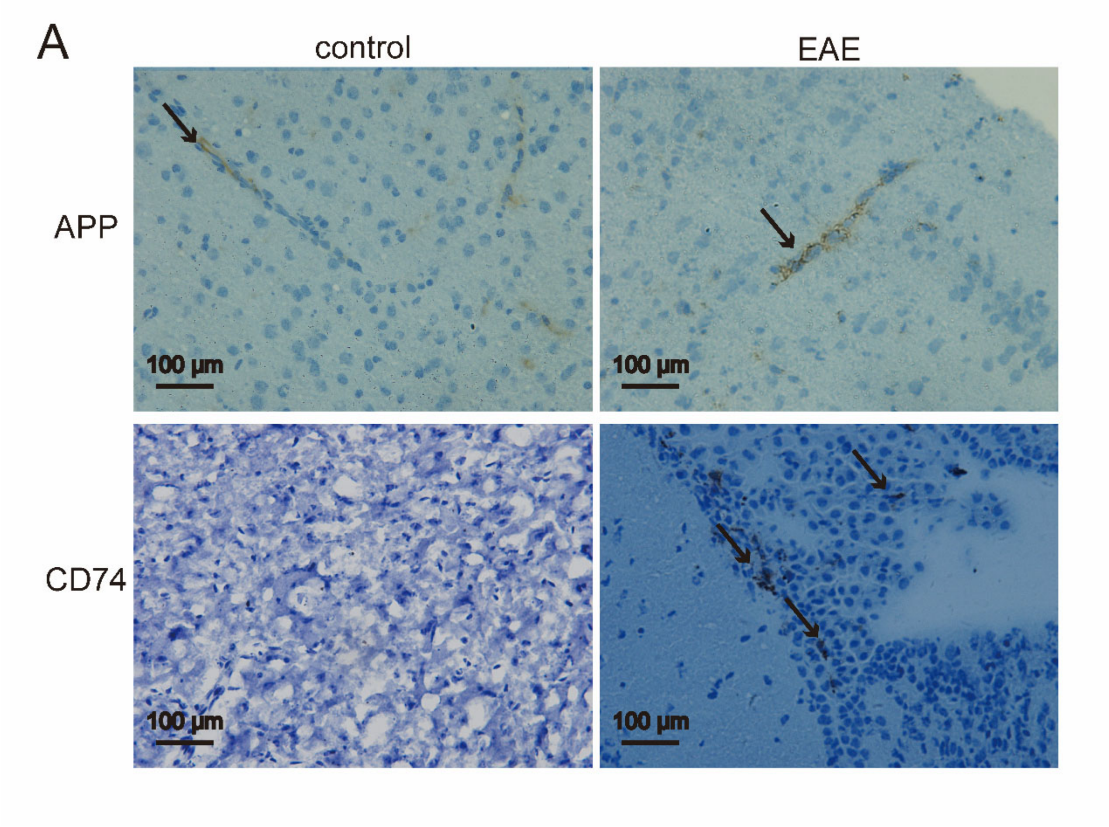

Supplementary Figure S7. (A) Immunohistochemical staining of APP and CD74 in brain tissues of the control group and EAE mice.

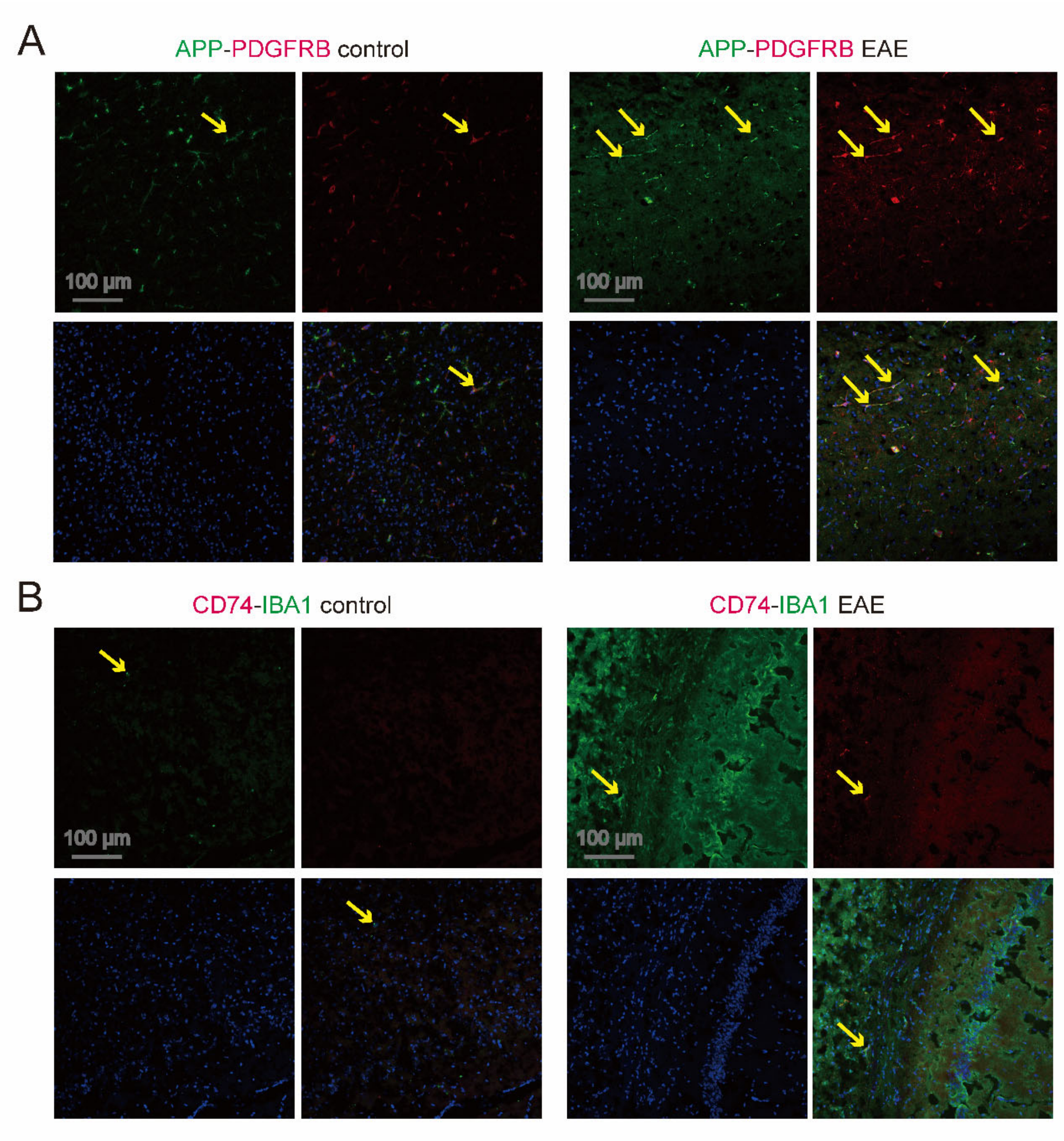

Supplementary Figure S8. (A) Immunofluorescence staining of APP and PDGFRβ in brain tissues of the control group and EAE mice. (B) Immunofluorescence staining for CD74 and IBA1 in brain tissues of the control group and EAE mice.

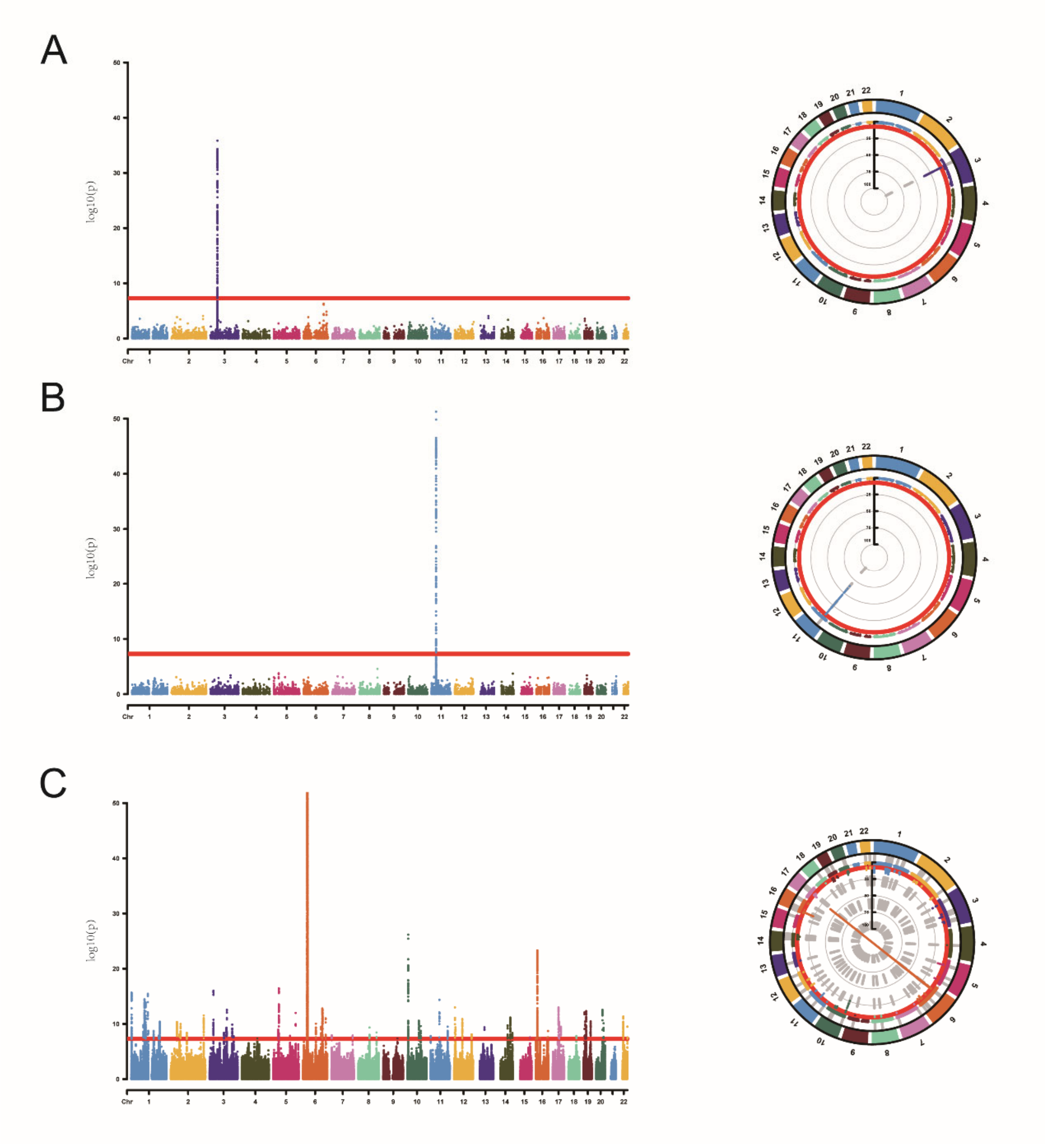

Supplementary Figure S9. (A) Manhattan plot and circular plot of *GPX1*. (B) Manhattan plot and circular plot of *RCN1*. (C) Manhattan plot and circular plot of MS.
